# Speech Timing Deficit of Stuttering: Evidence from Contingent Negative Variations

**DOI:** 10.1371/journal.pone.0168836

**Published:** 2017-01-09

**Authors:** Ning Ning, Danling Peng, Xiangping Liu, Shuang Yang

**Affiliations:** 1 School of Education, Soochow University, Suzhou, Jiang Su, China; 2 State Key Laboratory of Cognitive Neuroscience and Learning, Beijing Normal University, Beijing, China; 3 School of Psychology, Beijing Normal University, Beijing, China; Duke University, UNITED STATES

## Abstract

The aim of the present study was to investigate the speech preparation processes of adults who stutter (AWS). Fifteen AWS and fifteen adults with fluent speech (AFS) participated in the experiment. The event-related potentials (ERPs) were recorded in a foreperiod paradigm. The warning signal (S1) was a color square, and the following imperative stimulus (S2) was either a white square (the Go signal that required participants to name the color of S1) or a white dot (the NoGo signal that prevents participants from speaking). Three differences were found between AWS and AFS. First, the mean amplitude of the ERP component parietal positivity elicited by S1 (S1-P3) was smaller in AWS than in AFS, which implies that AWS may have deficits in investing working memory on phonological programming. Second, the topographic shift from the early phase to the late phase of contingent negative variation occurred earlier for AWS than for AFS, thus suggesting that the motor preparation process is promoted in AWS. Third, the NoGo effect in the ERP component parietal positivity elicited by S2 (S2-P3) was larger for AFS than for AWS, indicating that AWS have difficulties in inhibiting a planned speech response. These results provide a full picture of the speech preparation and response inhibition processes of AWS. The relationship among these three findings is discussed. However, as stuttering was not manipulated in this study, it is still unclear whether the effects are the causes or the results of stuttering. Further studies are suggested to explore the relationship between stuttering and the effects found in the present study.

## 1 Introduction

Stuttering is manifested by involuntary hesitations, interruptions, prolongations and repetitions of phonemes during speech [1]. To produce a word, people first generate a speech program, which includes the phonological and phonetic encoding processes then, they initiate the articulatory motor process [2]. It is argued that stuttering might occur when either process is advanced or delayed [3]. Many studies have shown that adults who stutter (AWS) have deficits in phonological encoding and speech motor control, but few have observed the dynamic interaction of the two processes during speech preparation.

Because speech preparation occurs rapidly, researchers usually use a foreperiod (FP) paradigm in a laboratory to magnify this process [4]. In this paradigm, the FP is the time interval between a warning stimulus (S1) and a following imperative stimulus (S2), which requires motor response[5]. Typically, the S1 evokes a sequence of event-related potentials (ERP), such as S1-N1, S1-P2, S1-N2, S1-P3, and a slow negative potential (i.e., the contingent negative variation, CNV) [6]. The CNV has at least two phases. The early phase presents maximum activity over the frontal sites and has generators in the supplementary motor area (SMA) and the anterior cingulate cortex (ACC) [7, 8]; it appears to reflect orientation, attention and temporal expectation [9, 10]. The late phase usually has a central-parietal distribution with origins in the pre/primary motor cortex, SMA, posterior parietal and secondary sensory cortex; it is related to advanced motor/sensory preparation for coming movements [8, 11].

Early studies using the FP paradigm to investigate the speech preparation process of AWS concentrate on the laterality of the late CNV [12–18]. However, the results were inconsistent. Achim, Braun and Collin (2007) noted that the “left hemisphere under-activation and right hemisphere over-activation” pattern of the late CNV might only relate to dysfluent speech. In a recent case study on acquired stuttering, Vanhoutte et al. (2014) found that the amplitude of the late CNV, which should reflect the degree of speech motor preparation, was inversely proportional to stuttering frequency. Later, Vanhoutte et al. (2015) observed a positive correlation between the slope of the late CNV and stuttering frequency/severity in adults with developmental stuttering. They argued that the abnormal CNV they obtained in AWS reflected the malfunctioning of the basal ganglia-thalamo-cortical network in stuttering.

Prescott and Andrews (1984) were the first to separately examine the early and late CNVs, but they found no significant group difference in the two CNV phases. This line of study was limited until 2000, when a magnetoencephalography (MEG) study revealed that AWS and adults with fluent speech (AFS) had different time sequences of brain activations [19]. They found that within the first 400 ms of speech preparation, AFS showed earlier activation in the left frontal lobe (articulatory programming) than in the left premotor area (motor preparation), whereas AWS exhibited the opposite pattern. The result implies that the motor process of AWS might be initiated too early, that is, before the articulatory program is ready. To test this hypothesis, a group of researchers examined the optimal FP (the FP that would lead to the fastest speech reaction time when the FP is fixed in a block) [20]. The results revealed that the optimal FP was approximately 200~400 ms for AWS and approximately 800 ms for AFS. The authors attributed the short optimal FP of AWS to the early initiation of the speech motor preparation process. They explained that if AWS were used to initiate the motor preparation process early at 200~400 ms, they would have difficulties in maintaining or improving their naming speed when the FP increased from 400 ms to 800 ms.

The optimal FP, although suggestive, is not a direct index of motor preparation. Studies found that the distribution of CNV is related to the momentary cognitive process [21]. More specifically, the distribution of CNV is more frontally located when people prepare to encode words into long-term memory, but it is more centrally distributed when the tasks are predominantly motor specific. Therefore, tracing the topographic shift of CNV may be a speculative approach to study the time course of motor preparation. The topographic characteristics of the early and late CNVs have been studied in patients with schizophrenia [22], migraines [23] and dementia [24]. However, these studies only employed a one-time window for each CNV phase, and therefore, the topographic shift between the two phases was not observed. In the present study, we compared the topographic features of the CNV interval-by-interval between AWS and AFS [8, 21] and examined whether the topographic shift of the CNV from the early to the late phase occurred earlier for AWS than for AFS.

To obtain a complete picture of the speech preparation process, the ERP components evoked by the S1 and S2 were also examined. Among the ERP components evoked by S1, we were most interested in the parietal P3 (S1-P3) because a larger S1-P3 amplitude is associated with a greater investment in working memory and better performance [25], and the relationship between working memory and stuttering has long been discussed [26–28]. Maxfield et al. (2005) reported a larger P280, which exhibited a similar latency and topographic distribution to S1-P3, in AWS when S1 (the cue) provided no phonological information about S2 (the picture to name). They suggested that AWS lacked focal attention during speech preparation. Hence, a weak S1-P3 is expected if AWS have difficulties putting enough working memory resources into speech preparation.

For the S2-evoked potentials, a Go/NoGo task was incorporated into the FP paradigm. Thus, there were two types of S2: one type (the Go signal) permitted participants to speak, and the other type (the NoGo signal) prevented them from speaking. The NoGo S2-N2 and S2-P3 are the indices of inhibitory control ability [29, 30]. The inhibitory control ability of people who stutter has only been tested in children by using the Amsterdan Neuropsychological task (a speech-unrelated Go/NoGo task) [31, 32]. The results were controversial. A behavioral study found that children who stutter had more false alarms in the Go/NoGo task [31], but an ERP experiment found no group difference in the NoGo S2-N2 and S2-P3 [32]. Considering that task complexities and the age of participants can influence the neural responses in the Go/NoGo tasks [29, 30, 33], the inhibitory control ability of AWS is still unclear. The third aim of this study was to preliminarily test the inhibitory control ability of speech for AWS.

## 2 Materials and Methods

### 2.1 Participants

The study was approved by the Ethics Committee of Soochow University. All participants provided a written informed consent to participate in this study. Fifteen AWS (2 females, mean age = 28.5 years, SD = 9.8) and fifteen AFS (2 females, mean age = 27.8 years, SD = 7.9) participated in the experiment (Table 1). The AWS were recruited from the Stuttering Association of China. All AWS began to stutter before they were seven-years-old and were diagnosed as mild to severe stutterers based on the Stuttering Severity Instrument, third edition (SSI-3) [34]. The mean severity score was 23.9 (SD = 4.7), ranging from 18 (mild) to 35 (severe). The AFS reported no history of speech impairments. All participants were native Mandarin speakers, righted-handed [35], had normal color vision [36], and had no history of hearing problems or neurologic or psychiatric disorders. The two groups were matched according to their gender, age (*t* (28) = 0.23, *p* > 0.05), and level of education (*χ*^2^ (2) = 0.48, *p* > 0.05).

**Table 1:** Participants, biographical data.

Id	Group	Gender	Age	Academic degree	Stuttering severity, SSI-3	
					Percentile	Category
1	AWS	M	31	B	21	mild
2	AWS	F	19	B	18	mild
3	AWS	M	30	D	24	moderate
4	AWS	M	19	B	22	mild
5	AWS	M	40	B	27	moderate
6	AWS	M	20	B	20	mild
7	AWS	M	18	B	29	moderate
8	AWS	M	21	B	23	moderate
9	AWS	M	29	D	19	mild
10	AWS	F	18	B	23	mild
11	AWS	M	22	B	20	mild
12	AWS	M	38	M	30	moderate
13	AWS	M	46	B	35	severe
14	AWS	M	43	M	22	mild
15	AWS	M	34	M	25	moderate
1	AFS	M	19	B		
2	AFS	M	32	M		
3	AFS	M	22	B		
4	AFS	M	36	B		
5	AFS	M	38	B		
6	AFS	F	23	M		
7	AFS	M	18	B		
8	AFS	M	22	B		
9	AFS	M	29	M		
10	AFS	M	28	B		
11	AFS	M	41	B		
12	AFS	M	36	M		
13	AFS	M	19	B		
14	AFS	F	20	B		
15	AFS	M	34	D		

Note. Gender: M = male; F = female.

Academic degree: B = Bachelor’s degree; M = Master’s degree; D = Doctor’s degree.

### 2.2 Design and Procedure

Participants were seated in a comfortable armchair and wore a Neuroscan QuikCap with 32 channels. A microphone was set 5 cm in front of the participant’s mouth to record their voice responses. Stimuli were presented with black background via a 14” monitor from a distance of approximately 140 cm. The experiment was controlled by the E-Prime 2.0 software (http://www.pstnet.com/products/e-prime/).

A color naming Go/NoGo task was applied. The NoGo trials accounted for one-fourth of the total trials. The experiment included 10 experimental blocks. Each block consisted of 24 Go trials and 8 NoGo trials, and their order was randomly arranged. In each trial, a color square (S1, visual angle = 1.6°×1.6°) was presented for 100 ms and then replaced by the black screen. After 1500 ms (the FP duration was 1600 ms), a Go/NoGo stimulus (S2, visual angle = 0.4°×0.4°) was presented for 1000 ms. The Go stimulus, which was a white triangle, required the participant to name the color of S1 with one syllable as soon as possible. The NoGo stimulus was a white dot, to which the participant was required to make no response. The response window was 3000 ms, and the inter-trial interval (ITI) varied randomly between 2800 ms and 3600 ms. The naming onset time was recorded when a voice response triggered a voice key during the response window. The color of S1 was randomly selected from four color choices (i.e., red, yellow, blue and green). Twelve trials of practice blocks were administered before the formal experiment.

### 2.3 EEG recording and analysis

EEGs were recorded at the sites of FP1/FP2, F7/F8, F3/F4, FZ, FT7/FT8, FC3/FC4, FCZ, T7/T8, C3/C4, CZ, TP7/TP8, CP3/CP4, CPZ, P7/P8, P3/P4, PZ, O1/O2, and M1/M1 according to the International 10–20 system with reference to the left mastoid. Horizontal and vertical electro-oculographs (HEOG and VEOG) were recorded with two bipolar electrodes attached laterally to the outer canthi of the eyes and 1 cm above and below the left eye. Electrode impedances were kept below 5kΩ. The EEG and EOGs were amplified (Synamps2, Neuroscan) with a sampling rate of 500 Hz and low-pass filtered at 100 Hz.

The EEGs were first transformed offline to an average reference of unlinked bilateral mastoids and filtered with a 30 Hz low pass cut-off. The VEOG artifacts were removed from the data by applying an eye-movement correction algorithm (Semlitsch et al., 1986). Then, the EEGs were segmented with time-locked to S1 and S2, respectively. For the S1-evoked ERPs, the EEGs were segmented into epochs of 1800 ms with 200 ms prior to the S1 as baseline. As there is no accepted method for removing the possible overlap of the CNV resolution with S2-evoked potentials, we simply used the 100 ms prior to the S2 as baseline for comparison with other studies [32]. Therefore, with respect to the S2-evoked ERPs, the EEGs were segmented into epochs of 1100 ms with 100 ms prior to the S2 as baseline. Any epochs containing artifacts or horizontal eye movements exceeding 100 μV in any channel were automatically rejected. Trials with incorrect responses were also rejected from further analysis.

### 2.4 Data analysis

#### 2.4.1 Behavioral data

For each participant, the naming accuracy (ACC) and the mean reaction time (RT) of correct responses in the Go trials, and the percentage of false alarms (FA) to the NoGo stimulus were computed. Naming on each trial was correct if the participant named the color square correctly and fluently within the response window. The disfluent responses, which were defined as whole-word substitutions, phonological errors or multi-word responses, were judged and counted offline by an experienced research assistant [27]. The ACCs and FAs were transformed into arcsin values. Independent sample *t*-tests were used to compare the mean RTs, transformed ACCs and FAs between the two groups. The cohen’s *d* was used for effect size estimation in all *t*-tests of the present study.

#### 2.4.2 S1-evoked potentials

Considering that the occurrence of S2 could not be predicted, the S1-evoked ERPs were averaged by collapsing the Go and the NoGo trials for each participant. Four S1-evoked components (i.e., S1-N1, S1-P2, S1-N2 and S1-P3) were recognized and quantified with baseline-to-peak amplitudes and latencies. For peak detection, a computer algorithm selected the maximum (for S1-P2 and S1-P3) or minimum (for S1-N1 and S1-N2) within a fixed latency range at a midline channel that is traditionally used to measure each component [29]. For both groups, the S1-N1 was searched at Fz in the time window of 50–170 ms, the S1-P2 was searched at Pz in the time window of 90–320 ms, the S1-N2 was searched at Fz in the time widow of 180–320 ms, and the S1-P3 was searched at Pz in the time window of 240–600 ms. Then, amplitude measurements were performed at the same latency at all other sites [37].

The statistical analysis of amplitudes was restricted to the midline sites used in peak detection and two adjacent sites on the left and right (i.e., F3/Fz/F4 for S1-N1, P3/Pz/P4 for S1-P2, F3/Fz/F4 for S1-N2, and P3/Pz/P4 for S1-P3). Separate repeated measure ANOVAs in a 3 (*Lateral*: left, midline and right) × 2 (G*roup*: AFS and AWS) matrix was applied to the amplitudes for each component. Planned contrasts for the *Lateral* factor compared the left with the right hemisphere (L vs. R), and the mean of these with the midline (Hemisphere vs. Midline). As these contrasts were planned and no post-hoc comparison was applied, the Bonferroni-type adjustment to alpha was not necessary [38]. The partial Eta squared was used for effect size estimation in all ANOVAs of the present study. As the latency of each component was locked at either Fz or Pz in the analyses, the latencies between the two groups were compared by independent sample *t*-tests.

#### 2.4.3 CNVs

Two approaches were used to analyze the CNV data. First, we explored the topographic shift between early and late CNVs by comparing the activations at frontal and central sites [8]. The course of the CNV was traced by computing the mean amplitude of each successive 100 ms interval from 400 ms to 1600 ms after S1. Nine electrodes (F3/Fz/F4, FC3/FCz/FC4, and C3/CZ/C4), which cover the frontal-to-central area, were included in the statistical analysis. The mean amplitudes for each CNV interval were subjected to a repeated measures ANOVA with *Group* (AWS, AFS) as a between-subject factor and *Sagittal* [Frontal (F3, Fz, F4) × Frontal-Central (FC3, FCz, FC4) × Central (C3, Cz, C4)] and *Lateral* [Left (F3, FC3, C3) × Midline (Fz, FCz, Cz) × Right (F4, FC4, C4)] as within-subject factors. Planned contrasts for the *Sagittal* factor compared frontal with frontal-central activation (F vs. FC), and frontal-central with central activation (FC vs. C), and for the *Lateral* factor compared the left with the right hemisphere (L vs. R), and the mean of these with the midline (Hemisphere vs. Midline). Such contrasts were optimal for deriving information about the topographic distributions of each CNV interval [29]. The false discovery rate procedure was applied to *p*-values generated in each time-interval to correct for multiple comparisons [39].

Second, the slope analysis was applied to compare the CNV slopes in AFS and AWS. The procedure was similar to that in Vanhoutte et al.’s study (2015).The time window of interest (TOI) was defined as 500 ms preceding S2. The CNV slope for each participant was computed by subtracting the mean amplitudes of the first (-500 to -400 ms) and the last (-100 to 0 ms) 100 ms of the TOI. Nine electrodes (F3/Fz/F4, C3/Cz/C4, P3/Pz/P4) were included in statistical analysis. The slope data were subjected to a repeated measure ANOVA with *Group* (AWS, AFS) as a between-subject factor and *Sagittal* [Frontal (F3, Fz, F4) × Central (C3, Cz, C4) × Parietal (P3, Pz, P4)] and *Lateral* [Left (F3, C3, P3) × Midline (Fz, Cz, Pz) × Right (F4, C4, P4)] as within-subject factors. Planned contrasts for the *Sagittal* factor compared frontal with parietal activation (F vs. P), and the mean of these with the central activation (F/P vs. C), and for the *Lateral* factor compared the left with the right hemisphere (L vs. R), and the mean of these with the midline (Hemisphere vs. Midline). In addition, a Spearmen correlation was calculated between the CNV slope at Cz and the overall percentile score of the SSI-3.

#### 2.4.4 S2-evoked potentials

The S2-evoked ERPs were averaged separately for the Go and the NoGo trials. The S2-N2 and the S2-P3 were recognized and measured with baseline-to-peak amplitudes and latencies. For peak detection, a computer algorithm selected the greatest negativity in the range of 200–300 ms at Fz as the S2-N2, and the greatest positivity in the range 280–550 ms at Pz as the S2-P3. Then, amplitude measurements were performed at the same latency at all other sites [37].

Nine sites that are traditionally used in Go/NoGo studies (i.e., F3/Fz/F4, C3/Cz/C4, P3/Pz/P4) were included in the statistical analysis. The amplitudes of S2-N2 and S2-P3 were subjected to a 2 (*Condition*: Go and NoGo) × 3 *(Sagittal*: frontal, central and parietal) × 3 (*Lateral*: left, midline and right) × 2 (*Group*: AWS and AFS) repeated measure ANOVA respectively. Planned contrasts for the *Sagittal* factor compared frontal with central activation (F vs. C), and central with parietal activation (C vs. P), and for the *Lateral* factor compared the left with the right hemisphere (L vs. R), and the mean of these with the midline (Hemisphere vs. Midline). As the latency of each component was locked at either Fz or Pz in the analyses, the latencies of S2-N2 and S2-P3 were subjected to a 2 (*Condition*: Go and NoGo) × 2 (*Group*: AWS and AFS) repeated measure ANOVA separately.

## 3 Results

### 3.1 Behavior results

All participants were mainly fluent in the experiment. Only ten disfluent responses were found in two AWS. The ACCs were higher in AFS than in AWS, *t* (28) = -2.57, *p* < 0.05, *d* = 0.94 (Table 2). The mean RTs for the two groups were not significantly different, *t* (28) = 0.28, *p* > 0.05, *d* = 0.10, nor were the percentages of false alarms for the two groups, *t* (28) = -1.02, *p* > 0.05, *d* = 0.37.

**Table 2:** Mean RTs, ACCs, FAs and their standard deviations for AWS and AFS.

Group	ACC (%)	RT (ms)	FA (%)
**AWS**	98.87 (0.74)	576 (77)	5.33 (2.13)
**AFS**	99.47 (0.52)	567 (100)	4.60 (2.61)

### 3.2 S1 evoked ERPs

No group difference was found in the latencies of each S1-evoked potential (Table 3). Significant midline > hemisphere effects were observed in the amplitudes of all S1 evoked ERPs, S1-N1: *F*(1,28) = 18.87, *p* < 0.001, *η*^*2*^_*p*_ = 0.40; S1-P2: *F*(1,28) = 4.53, *p* < 0.05, *η*^*2*^_*p*_ = 0.14; S1-N2: *F*(1,28) = 20.23, *p* < 0.001, *η*^*2*^_*p*_ = 0.42; S1-P3: *F*(1,28) = 14.12, *p* < 0.01, *η*^*2*^_*p*_ = 0.34.

**Table 3:** Mean latencies and their standard deviations (ms) of each S1-evoked potential for AWS and AFS and the results of *t*-tests.

Component	AFS	AWS	*t (28)*	*p*	*Cohen’s d*
**S1-N1**	74 (20)	80 (26)	-0.73	0.473	0.27
**S1-P2**	178 (40)	187 (20)	-0.72	0.479	0.26
**S1-N2**	259 (27)	257 (26)	0.28	0.785	0.10
**S1-P3**	361 (98)	353 (77)	0.24	0.810	0.09

**Fig 1:**
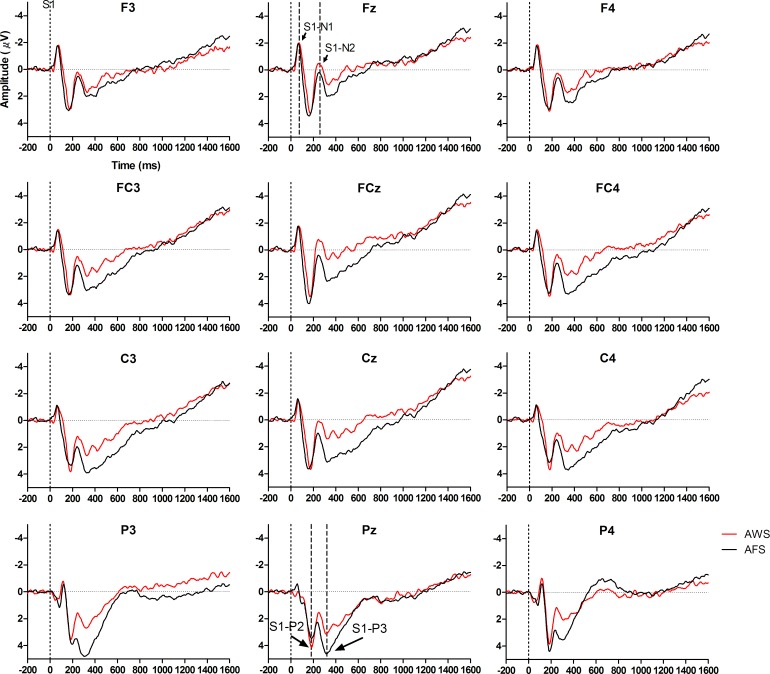
The S1-evoked ERPs for AWS (red line) and AFS (black line). The mean amplitudes of ERPs at different electrodes are presented. Examples of S1-N1 and S1-N2 are shown at Fz, and examples of S1-P2 and S1-P3 are shown at Pz.

**Fig 2:**
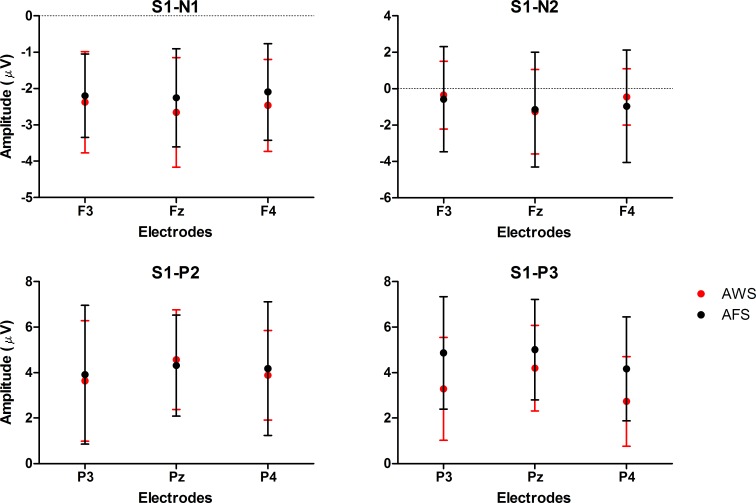
Peak amplitudes for AWS and AFS. The mean peak amplitudes and the standard deviations of S1-N2 and S1-P3 elicited by S1 are shown for AWS and AFS.

The S1-P3 was left lateralized; its amplitude was larger at P3 than at P4, *F*(1,28) = 10.31, *p* < 0.01, *η*^*2*^_*p*_ = 0.27. More importantly, a significant group main effect was found in the S1-P3; the mean amplitude of S1-P3 was smaller for AWS than for AFS, *F*(1,28) = 6.18, *p* < 0.05, *η*^*2*^_*p*_ = 0.18 (Figs 1 and 2).

### 3.3 CNVs

#### 3.3.1 The topographic shift of CNVs

**Fig 3:**
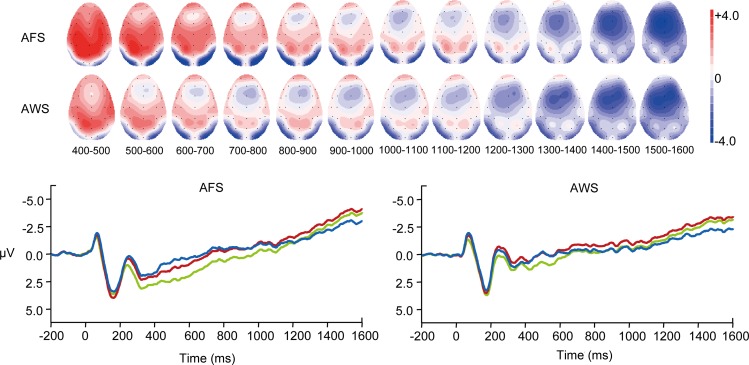
Topographic shifts of the CNV. The top panel presents the scalp voltage distribution of AFS and AWS, and the lower panel displays the mean amplitudes of ERPs at the FZ (blue line), the FCZ (red line) and the CZ (green line).

Approximately 400 ms after S1, the potential slowly shifted towards the negative pole, and its negative center moved from the frontal sites to the central sites. Though the magnitudes of the slow wave were slightly more negative for AWS than for AFS in the early phase, the differences were not significant. Interestingly, significant *Sagittal* (F vs. FC) × *Group* interactions in the two intervals between 500 and 700 ms indicated that the trend of this topographic shifting differed between the two groups (Fig 3 and Table 4). Regarding AFS, the negative center of the slow potential was still in the FZ between 500–700 ms; but for AWS, it began to spread into the FCZ between 500–600 ms, and moved to FCZ between 600–700 ms. It showed that the shifting point from the FZ to the FCZ occurred much earlier for AWS than for AFS.

**Table 4:** The Sagittal main effects and the *Sagittal* × *Group* interactions for each CNV interval.

CNV interval	*Sagittal*				*Sagittal* × *Group*			
	*F vs*. *FC*		*FC vs*. *C*		*F vs*. *FC*		*FC vs*. *C*	
	*F*	*η*^*2*^_*p*_	*F*	*η*^*2*^_*p*_	*F*	*η*^*2*^_*p*_	*F*	*η*^*2*^_*p*_
**400–500**	** 15.28	0.35	*** 35.16	0.56	4.20	0.13	0.00	0.00
**500–600**	** 15.24	0.35	*** 36.87	0.57	* 6.61	0.19	0.04	0.00
**600–700**	2.61	0.09	*** 18.06	0.39	* 6.79	0.12	0.17	0.01
**700–800**	0.53	0.02	** 14.56	0.34	5.96	0.18	0.07	0.00
**800–900**	0.14	0.01	** 13.97	0.33	5.08	0.15	0.12	0.00
**900–1000**	0.11	0.00	* 10.79	0.28	4.39	0.14	0.09	0.00
**1000–1100**	0.96	0.03	* 10.49	0.27	3.36	0.11	0.06	0.00
**1100–1200**	2.77	0.09	* 9.41	0.25	2.35	0.08	0.02	0.00
**1200–1300**	* 7.19	0.20	* 7.06	0.20	1.09	0.04	0.08	0.00
**1300–1400**	** 11.20	0.29	5.88	0.17	0.78	0.03	0.15	0.00
**1400–1500**	** 15.28	0.35	4.22	0.13	0.51	0.02	0.13	0.01
**1500–1600**	*** 19.34	0.41	3.24	0.10	0.41	0.01	0.12	0.00

Note.

* for *p* < 0.05.

** for *p* < 0.01.

*** for *p* < 0.001.

The CNV amplitudes between the left and right hemispheres were not found to be significantly different for both groups. No significant difference of laterality was found between the two groups, neither.

#### 3.3.2 The CNV slope

For both groups, the CNV slope showed a central-midline maximum, with a midline > hemisphere effect, *F*(1,28) = 23.82, *p* < 0.001, *η*^*2*^_*p*_ = 0.46, a C > F/P effect, *F*(1,28) = 98.68, *p* < 0.001, *η*^*2*^_*p*_ = 0.78, and a F > P effect, *F*(1,28) = 5.78, *p* < 0.05, *η*^*2*^_*p*_ = 0.17. A significant *Sagittal* × *Group* interaction showed that the midline > hemisphere effect was larger for AFS than for AWS, *F*(1,28) = 5.34, *p* < 0.05, *η*^*2*^_*p*_ = 0.16 (Table 5). The correlation between the CNV slope and stuttering severity was not significant, *r* = -0.24, *p* > 0.05.

**Table 5:** The mean slope values and standard deviations at the nine electrodes for AFS and AWS.

Group	Sagittal	Left	Midline	Right
**AFS**	**Frontal**	1.96 (1.18)	1.93 (1.38)	1.61 (1.27)
	**Central**	2.42 (1.37)	2.91 (1.60)	2.76 (1.30)
	**Parietal**	0.86 (0.90)	1.56 (1.19)	1.19 (1.04)
**AWS**	**Frontal**	1.31 (1.18)	1.40 (1.33)	1.12 (1.17)
	**Central**	1.76 (1.41)	1.95 (1.47)	1.70 (1.22)
	**Parietal**	0.70 (0.91)	1.12 (0.79)	0.79 (1.43)

### 3.4 S2 evoked Go/NoGo ERPs

#### 3.4.1 The S2-N2

The S2-N2 emerged at approximately 255 ms after the S2 with no differences between the two groups and conditions in the latencies (Table 6). The highest peak of N2 was at Fz, F > C: *F*(1,28) = 17.91, *p* < 0.001, *η*^*2*^_*p*_ = 0.39; C > P: *F*(1,28) = 23.96, *p* < 0.001, *η*^*2*^_*p*_ = 0.46; Midline > Hemisphere: *F*(1,28) = 14.83, *p* < 0.01, *η*^*2*^_*p*_ = 0.35. The S2-N2 was more left lateralized at the parietal sites than at the frontal sites, (C vs. P) × (L vs. R): *F*(1,28) = 9.97, *p* < 0.01, *η*^*2*^_*p*_ = 0.26. A significant *Group* × *Sagittal* (C vs. P) × *Lateral* (Midline vs. Hemisphere) interaction was observed, *F*(1,28) = 8.85, *p* < 0.01, *η*^*2*^_*p*_ = 0.24. It indicated that the Midline > Hemisphere effect of S2-N2 increased from the central sites to the parietal sites for AFS, but decreased or even inversed for AWS.

**Table 6:** Mean latencies and standard deviations (ms) of S-N2 and P3 for AWS and AFS.

Component	Go		NoGo	
	AFS	AWS	AFS	AWS
**N2**	256 (35)	254 (24)	253 (27)	257 (21)
**P3**	382 (64)	409 (64)	437 (54)	449 (36)

**Fig 4:**
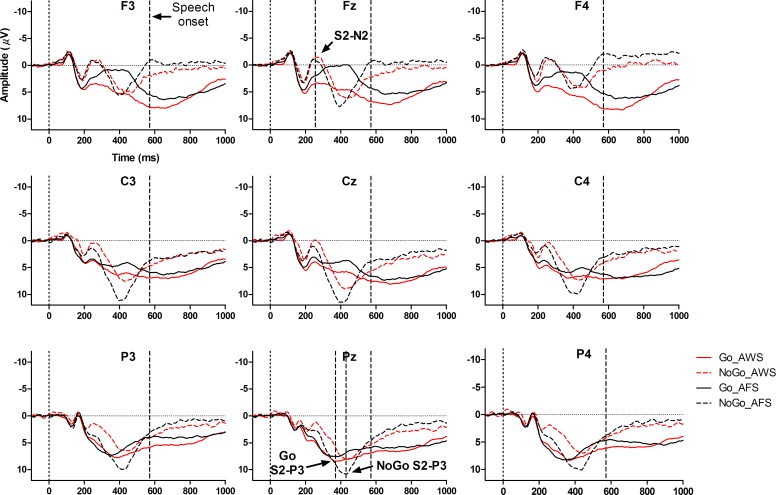
The S2-evoked ERPs. The mean amplitudes of ERPs at different electrodes are presented for AWS (red solid line) and AFS (black solid line) under the Go condition and for AWS (red dashed line) and AFS (black dashed line) under the NoGo condition. The mean RTs (speech onset time) are indicated in vertical dashed lines. An example of S2-N2 is shown at Fz, and an example of S2-P3 is shown at Pz.

**Fig 5:**
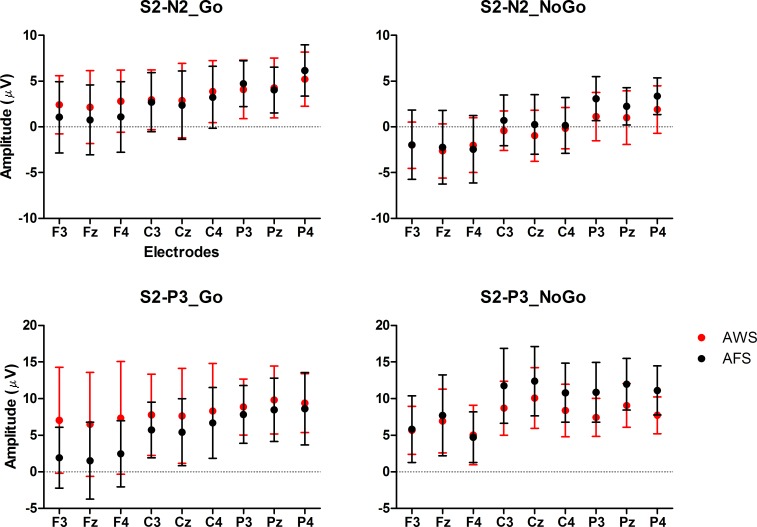
Peak amplitudes for AWS and AFS. The mean peak amplitudes and the standard deviations of S2-N2 and S2-P3 elicited by the Go and the NoGo signals are shown for AWS and AFS.

A significant NoGo > Go effect was found in the S2-N2 amplitude, *F*(1,28) = 55.55, *p* < 0.001, *η*^*2*^_*p*_ = 0.67 (Figs 4 and 5). This effect was larger at the frontal sites than at the central sites, *F*(1,28) = 7.49, *p* < 0.05, *η*^*2*^_*p*_ = 0.21, and also larger at the right sites than at the left sites, *F*(1,28) = 19.09, *p* < 0.001, *η*^*2*^_*p*_ = 0.41.

#### 3.4.2 The S2-P3

For both groups, the latencies and distributions of S2-P3 significantly differed between the two conditions. The S2-P3 reached its highest peak about 44 ms later in the NoGo condition than in the Go condition, *F*(1,28) = 11.64, *p* < 0.01, *η*^*2*^_*p*_ = 0.29 (Table 6). In the NoGo condition, the highest peaks of P3 were observed over the central and parietal area of the midline sites; but in the Go condition, the distribution of S2-P3 was concentrated over the parietal sites and more right-lateralized [Condition × Sagittal (F vs. C): *F*(1,28) = 12.98, *p* < 0.001, *η*^*2*^_*p*_ = 0.32; Condition × Sagittal (C vs. P) × Lateral (L vs. R): *F*(1,28) = 6.41, *p* < 0.05, *η*^*2*^_*p*_ = 0.19; Condition × Sagittal (C vs. P) × Lateral (Midline vs. Hemisphere): *F*(1,28) = 16.07, *p* < 0.001, *η*^*2*^_*p*_ = 0.37].

Importantly, a significant interaction was found between condition and group, *F*(1,28) = 4.58, *p* < 0.05, *η*^*2*^_*p*_ = 0.14 (Fig 4). Simple effect tests showed that the NoGo > Go effect was significant for AFS, *F*(1,28) = 7.64, *p* < 0.05, *η*^*2*^_*p*_ = 0.21, but not significant for AWS, *F*(1,28) = 0.07, *p* > 0.05, *η*^*2*^_*p*_ = 0.00. The distributions of S2-P3 were also found to be different between the two groups. The amplitudes of S2-P3 decreased greater from the central sites to the frontal sites for AFS than for AWS, *F*(1,28) = 9.49, *p* < 0.01, *η*^*2*^_*p*_ = 0.25.

## 4 Discussions

The time course of speech preparation is examined in AWS and AFS by using a modified FP paradigm. Following S1, the S1-N1, S1-P2, S1-N2, S1-P3 and CNV sequentially emerged. Three prominent differences were found between AWS and AFS. First, the amplitude of S1-P3 was significantly smaller in AWS than in AFS. Second, the topographic shift of the CNV from the frontal to the central areas occurred earlier in AWS than in AFS. Third, the NoGo S2-P3 effect (NoGo–Go) was larger for AFS than for AWS.

### 4.1 The CNV as an index of stuttering

The CNV emerges when people are preparing and waiting for a response. If AWS have deficits in speech preparation, there may be some indices of the CNV that distinguish AWS and AFS. Thus, the results of this study are compared with the results of previous studies in terms of those index candidates, i.e., the laterality, amplitude, slope and topography of CNVs.

AWS did not reveal different lateralizing patterns from AFS in the present experiment. A early study with a small size reported that four of five of AFS showed a larger CNV shift in the left hemisphere, but only 22% AWS showed the left-laterality tendency [18]. However, the results were not repeated by later studies [13–15]. One explanation for the consistent cerebral laterality of CNVs between AWS and AFS lies in the possibility that the right-lateralized pattern of the CNV is more closely related with stuttered responses[12]. To control the artifacts from motor responses in ERP studies on stuttering, the speech tasks, such as the color naming task in the present study, are usually easy. Therefore, the vocal responses would be mainly fluent, and ERPs with disfluent responses will be excluded from statistical analysis. Another explanation is that the over-activation of the right hemisphere is regarded as compensations for the low-efficiency of the left hemisphere [40]. Accordingly, the color naming task in the present study might be so easy for ASW that they do not need to involve more neural activities in the right hemisphere.

The amplitudes of the CNV were not significantly different between AWS and AFS, which is consistent with the results of most previous studies that directly compared the amplitudes of the CNV between AWS and AFS [12–18]. It seems that the amplitude of the CNV is not a sensitive measure for distinguishing between AWS and AFS.

With respect to the slope of the CNV, AWS did not exhibit a sharper late CNV in the present experiment; and the slope of late CNV was not correlated with stuttering severity. Because the task was different from that used in Vanhoutte et al.’s study (2015), more evidence is needed to test the reliability of this index.

The advanced topographic shift of the CNV in AWS found in this experiment implies that the early CNV is transient and the late CNV is promoted in AWS. The early CNV is found to be related to the timing of responses [41], and its generator (SMA) is involved in time estimation [40], linear sequence encoding of word production [41] and internal timing loops of AWS [40]. The transient early CNV is consistent with the hypothesis that AWS have deficits in speech timing. The late CNV correlates with motor preparation and has generators in the pre/primary motor cortex [8]. The early arrival of the late CNV is consistent with the early optimal FP for the naming response found in Ning et al.’s (2009) study [20]. Both imply that the motor preparation process is promoted in AWS. Additionally, the results support Selmelin et al.’s (2000) finding that the premotor cortex was activated ahead of time during speech preparation [19].

According to speech production models, a speech motor program is activated after phonological encoding [2]. If the motor preparation process is activated but the phonological program has not been generated yet, people should put more resources to maintain the high neural activating status of motor preparing, otherwise, the degrees of neural activation would decrease and upcoming motor responses would be influenced. The first case was not observed in the present study since the amplitudes of the ERPs during speech preparation were not found to be higher in AWS than in AFS. The second case is supported by a MEG study, which found that the brain activity before voluntary speech movement was lower for AWS than for AFS [28]. According to the EXPLAN theory proposed by Howell and Au-Yeung (2002), if the motor preparation process is promoted but the phonological program has not finished yet, people may try to compensate for the mismatching by inserting a pause or a meaningless sound (i.e. /a/), repeating the first phoneme, or prolonging the first phoneme then, stuttering occurs. Therefore, the present results support the view that the promoted speech motor preparation is one of the proximal contributor of stuttering [1].

### 4.2 The S1-P3

The S1-P3 obtained by using the FP paradigm is related to the coordination of the stimulus processing of S1 and the response preparation of S2 [42]. A larger S1-P3 amplitude was usually associated with a better response to S2 [25, 43]. This is confirmed by the results of the present study, as both the amplitude of S1-P3 and the response accuracy were lower for AWS than for AFS.

In addition to the working memory hypothesis, the S1-P3 is also proposed to reflect the process when people retrieve a well-established stimulus-response mapping for a response [42]. This account was echoed by a study on stuttering. Maxfield et al. (2015) found that the P280 (a morphologically similar ERP component to the S1-P3) was lower in AWS, but not in AFS, when the S1 contained phonological information about S2 [27]. The authors explained that P280 indexed the process of enhancing focal attention to facilitate the retrieval of specific information, and this process was attenuated in AWS when the phonological information about S2 was provided in advance. AWS have been reported to have difficulties in the phonological memory and the phonological encoding processes [26]. Taken together, the weaker amplitude of S1-P3 in the present paper might reflect the deficit of AWS in investing enough working memory to form a well-established phonological program for an upcoming response.

### 4.3 Response inhibition

The Go/NoGo task directly tests the inhibitory ability of an initial prepared response. In the present study, the group difference of the NoGo effect was found in the S2-P3, but not in the S2-N2 and the number of false alarms. Smith Johnstone, and Barry (2007) found that the S2-P3 was more sensitive in detecting response inhibition than the S2-N2 [30]. The smaller NoGo effect of AWS in S2-P3 in the present study suggests that AWS have difficulties in inhibiting a planed speech response.

The present results did not accord with those of previous studies in children who stutter. Egger et al. (2013) found that children who stutter have more false alarms than children who do not stutter under the Go/NoGo Task. But in an ERP study, Piispala, Bloigu, and Jansson-Verkasolo (2016) did not find group difference of the NoGo effect [32]. They only observed longer S2-N2 and S2-P3 latencies in children who stutter than in children who do not stutter, and suggested that children who stutter had deficits in attentional processing such as stimulus evaluation and response selection rather than in inhibitory control. One possible explanation is attributed to the inconsistent findings that the neural correlates of inhibitory control function differently for children than they do for adults [33]. This is especially true for the striatal activity, which is aberrant in many neural image studies on stuttering [31]. Moreover, striatal activity was found to be supportive and contributory to cognitive control in children but obstructive in adults. Accordingly, the brain correlates of stuttering might not coincide with those of cognitive control for AWS. Another reason for the inconsistent results is that the task (i.e., color naming) we used is more challenging for AWS than the button pressing task used in the two studies above [31, 32]. As the S2-P3 is sensitive to task difficulties, the deficits of inhibitory control abilities of AWS might be more prominent in speech-related tasks [43].

### 4.4 Caveats, limitations and suggestions for future research

The most important findings for AWS in the present study are the low amplitude of S1-P3 and the advanced topographic shift from the early CNV to late CNV. The former points to a low investment of working memory on phonological encoding, and the latter implies an aberrant timing of speech response. However, one should be cautious when considering their relationship. The S1-P3 and the CNV can be simultaneously influenced by certain factors, such as learning [43], but they can also be dissociated by different factors, such as monetary rewards [25]. Hence, it is too early to say whether the atypical speech motor preparation process of AWS is caused by their low investment in working memory or whether the two deficits just coincidentally appear together. More elaborately designed studies are needed to address this question. And more advanced neuro-image skills with high spatial-resolution are needed to describe the dynamic brain activations during speech preparation for both AWS and AFS.

The second concern is about the NoGo S2-P3 effect in AWS. As an index of inhibitory control, the NoGo S2-P3 effect is not independent, but influenced by the information processing of S1 during FP. For instance, the NoGo S2-P3 effect is found to increase when S1 provided more information about S2 [30]. And some of the neural correlates of inhibitory control overlap with the cortical-basal ganglia network. The latter is suspected to be related to the timing of speech and play an important role in stuttering [40]. In the present study, the lower parietal S1-P3 for AWS indicates that level of information processing of S1 is lower for AWS than for AFS. Then it is possible that the smaller NoGo S2-P3 effect in AWS is caused by their atypical speech preparation process. However, to clearly examine the causal relationship between the S1-P3 and the S2-P3 in AWS, further studies are needed to dissociate the speech preparation process and the inhibitory control abilities of AWS.

Lastly, this study is a quasi-experiment; stuttering was not manipulated. Although the differences between AWS and AFS in the three indices ((i.e., the S1-P3, the topographic shift of CNV, and the S2-P3) suggest that AWS have deficits in the investment of working memory on phonological encoding, the timing of speech, and the inhibitory control of speech, it’s still unclear whether they are the causes or the effects of stuttering. Studies that compare the neural activities of AWS before and after treatments should pay more attention to evaluate the speech preparation process.

## Supporting Information

S1 File Data for S1-evoked potentials. (XLS)

S2 File Data for CNVs. (XLS)

S3 File Data for S2-evoked potentials. (XLS)
